# Novel urine cell-free DNA methylation markers for hepatocellular carcinoma

**DOI:** 10.1038/s41598-023-48500-y

**Published:** 2023-12-07

**Authors:** Selena Y. Lin, Wei Xia, Amy K. Kim, Dion Chen, Shelby Schleyer, Lin Choi, Zhili Wang, James P. Hamilton, Harry Luu, Hie-Won Hann, Ting-Tsung Chang, Chi-Tan Hu, Abashai Woodard, Terence P. Gade, Ying-Hsiu Su

**Affiliations:** 1 JBS Science, Inc., Doylestown, PA USA; 2 Division of Gastroenterology and Hepatology, Department of Medicine, The Johns Hopkins University School of Medicine, Baltimore, MD USA; 3 ClinPharma Consulting, Inc., Phoenixville, PA USA; 4 Department of Medicine, Division of Gastroenterology and Hepatology, Thomas Jefferson University Hospital, Philadelphia, PA USA; 5 Department of Internal Medicine, National Cheng Kung University Hospital, College of Medicine, National Cheng Kung University, Tainan, Taiwan; 6 Division of Gastroenterology, Department of Internal Medicine, Hualien Tzu-Chi Hospital, Buddhist Tzu-Chi Medical Foundation, Hualien, Taiwan; 7 Department of Radiology, University of Pennsylvania College of Medicine, Philadelphia, PA USA; 8 The Baruch S. Blumberg Institute, 805 Old Easton Rd, Doylestown, PA USA

**Keywords:** Cancer screening, Cancer epigenetics

## Abstract

An optimized hepatocellular carcinoma (HCC)-targeted methylation next generation sequencing assay was developed to discover HCC-associated methylation markers directly from urine for HCC screening. Urine cell-free DNA (ucfDNA) isolated from a discovery cohort of 31 non-HCC and 30 HCC was used for biomarker discovery, identifying 29 genes with differentially methylated regions (DMRs). Methylation-specific qPCR (MSqPCR) assays were developed to verify the selected DMRs corresponding to 8 genes (*GRASP*, *CCND2*, *HOXA9*, *BMP4*, *VIM*, *EMX1*, *SFRP1*, and *ECE)*. Using archived ucfDNA, methylation of *GRASP*, *HOXA9*, *BMP4*, and *ECE1*, were found to be significantly different (*p* < 0.05) between HCC and non-HCC patients. The four markers together with previously reported *GSTP1* and *RASSF1A* markers were assessed as a 6-marker panel in an independent training cohort of 87 non-HCC and 78 HCC using logistic regression modeling. AUROC of 0.908 (95% CI, 0.8656–0.9252) was identified for the 6-marker panel with AFP, which was significantly higher than AFP-alone (AUROC 0.841 (95% CI, 0.778–0.904), *p* = 0.0026). Applying backward selection method, a 4-marker panel was found to exhibit similar performance to the 6-marker panel with AFP having 80% sensitivity compared to 29.5% by AFP-alone at a specificity of 85%. This study supports the potential use of methylated transrenal ucfDNA for HCC screening.

## Introduction

Hepatocellular carcinoma (HCC) is the third leading cause of cancer deaths^1,2^. It is often detected at late stages with a dismal five-year survival rate of 17.6%^3^ even with the implementation of HCC screening in a well-defined at-risk population. Early detection can improve prognosis when curative treatments are implemented^4–6^. Unfortunately, the current standard-of-care for HCC screening, ultrasound alone or with serum alpha feto-protein (AFP), has a poor sensitivity of 40% for detecting early HCC. In addition to serum AFP, the markers fucosylated AFP-L3% and serum DCP/PIVKA-II are used as HCC risk markers. As none of these risk markers have sufficient sensitivity alone (40–60% sensitivity) for HCC screening^7–9^, they are recommended to be used with ultrasound^10^ to identify patients from at-risk populations to undergo evaluation by MRI/CT imaging for HCC diagnosis. Providing a convenient, noninvasive, and sensitive approach such as genetic liquid biopsies to detect more HCC at early stages is one approach to improve patient outcomes.

Urine has been shown by us and others to be a reliable source for cell-free DNA (cfDNA) for cancer screening and monitoring^11–18^. Epigenetic alterations such as increased DNA methylation levels in critical genes can signify early tumorigenesis events presenting an opportunity for early cancer detection^19^. In a recent multicenter blinded cohort study (n = 609)^20^, a panel of urine circulating tumor DNA (ctDNA) markers was selected and developed using HCC-associated DNA modifications, mutated *TP53* gene and two methylated DNA markers *GSTP1 (mGSTP1)* and *RASSF1A (mRASSF1a)* for HCC screening. The performance of these three ctDNA risk markers with serum AFP, showed great promise detecting 30% more HCC as compared to serum AFP alone including earlier stage HCC. The performance of this urine ctDNA panel led us to investigate additional methylated gene targets to add to our current 3-ctDNA panel to improve the performance of the current 3 ctDNA panel urine test. We envision this test can be used alone or with the current HCC screening guidelines. Our recent study of profiling cfDNA between matched urine and plasma showed that the composition of cfDNA in urine is not the same as in plasma^21^, therefore, to discover HCC urine ctDNA markers, one should directly use urine cfDNA over DNA isolated from plasma or HCC tissue. Currently, there is no HCC risk or diagnostic marker available via next generation sequencing of cfDNA in urine.

One of the challenges in using urine ctDNA is its low quantity and high fragmentation^22,23^. Furthermore, high background DNA from renal and postrenal origins can contribute to the difficulty of ctDNA marker discovery in urine. In this HCC urine biomarker discovery study, a methyl-seq NGS assay suitable for extensively fragmented DNA^24,25^ for known HCC associated methylation genes^26^ was optimized and used to develop a discovery cohort to detect HCC-associated methylation sites. This was followed by methylation-specific quantitative PCR (MSqPCR) validation for selected HCC-differential methylation regions and further biomarker development using a training set. A panel of 4 and 6 urine methylation markers were developed for validation providing an indication of its potential for noninvasive cancer screening. This is the first study utilizing urine DNA to discover transrenal methylation DNA biomarker and provides evidence for its use in noninvasive cancer screening.

## Results

### Urinary methylated HCC biomarker discovery by methyl-seq NGS assay

**Figure 1:**
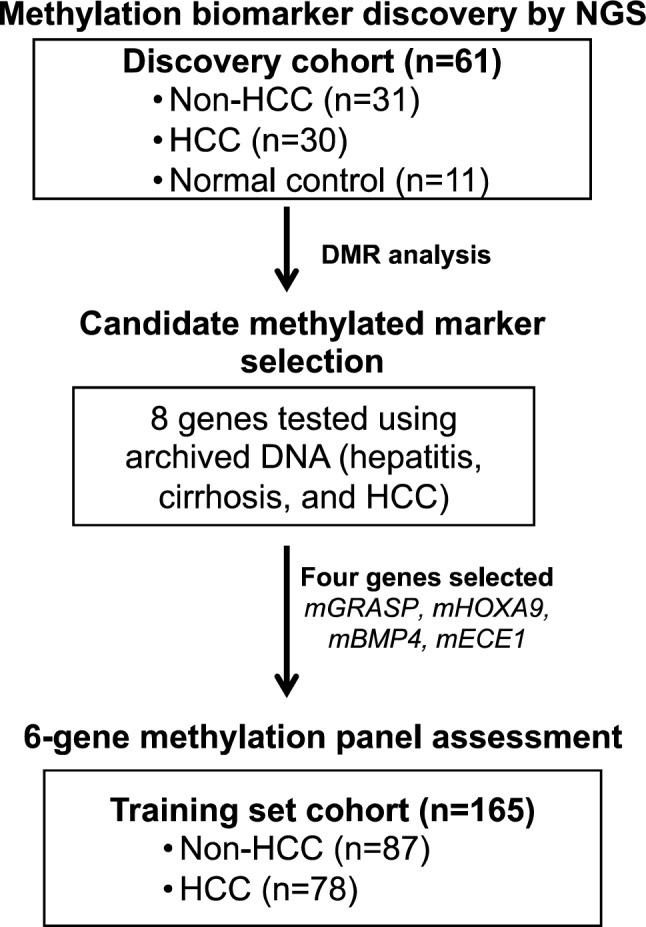
Flow diagram showing outline of the study.

To discover methylated DNA markers for HCC screening directly from urine, as outlined in Fig. 1, methyl-seq NGS assay was performed on ucfDNA from the discovery cohort comprised of at-risk non-HCC (HBV-hepatitis and cirrhosis) vs. HCC patients (Table 1). To minimize the effect of age-associated methylation changes on some CpG sites^27^, the patient samples in the two groups were closely matched for age. The total number of genes identified by differentially methylated region (DMR) analysis at a bin size of 1:200:1 were 29 genes including our previously identified urine HCC marker, *mRASSF1a* (Table 2). To verify the results from methyl-seq by MSqPCR and further develop candidates to be urinary HCC biomarkers, the most promising gene DMRs were selected for assay development that have > 0.005 meth-diff, which is the estimated methylation difference between HCC and non-HCC and included at least 4 CpG sites in a target region of 70 bp or less. Two examples shown in Supplementary Fig. 1 illustrated these selection criteria. A 339 bp DMR in *RSPH9* did not contain 4 CpG sites with sufficient specificity (> 0.005 meth-diff) as shown in Supplementary Fig. 1A. An example of a DMR selected for further development is *EMX1*. The 386 bp DMR in EMX1 has two regions with ≥ 0.005 meth-diff between non-HCC and HCC and with more than 4 CpG sites positioning within 70 bp (Supplementary Fig. 1B). Based on these criteria, eight DMR gene regions, *GRASP*, *CCND2*, *HOXA9*, *BMP4*, *VIM*, *EMX1*, *SFRP1*, and *ECE1* were selected*,* from which a short-amplicon MSqPCR was developed accordingly. The total number of targeted CpG sites per assay and the MSqPCR assay condition are summarized in Supplementary Table 1 with the assay detection limit and linearity shown in Supplementary Fig. 2.

**Table 1:** Patient clinical characteristics of the pilot cohort in this study.

Diagnosis	Patient cohort (n = 61)	
	Non-HCC (n = 31)	HCC (n = 30)
Median age (IQR range), years	57.0 (36–79)	61.0 (41–72)
Gender (M:F)	15:16	23:7
Etiology		
HBV	19	1
HCV	2	13
Non-viral	10	16
BCLC Stage (n)		
0		0
A		9
B		14
C		6
D		1
AFP (ng/mL)		
< 20	29	21
≥ 20	2	8
NA	0	1

BCLC: Barcelona Clinic Liver Cancer staging. AFP: alpha fetoprotein; HBV: Hepatitis B, HCV: Hepatitis C; NA, not available.

**Table 2:** Differentially methylated regions identified in urine cfDNA between non-HCC (n = 31) and HCC (n = 30) patients.

Chr	Start	End	Region Size	CpG sites	Meth-diff	Gene
chr1	21,616,786	21,616,898	112	3	0.00833724	ECE1_2
chr1	25,256,226	25,256,949	723	25	0.0140813	RUNX3_2
chr1	171,810,750	171,810,901	151	2	0.00641421	DNM3
chr1	171,810,918	171,810,974	56	1	0.00853242	DNM3
chr2	29,033,698	29,033,998	300	4	0.0136426	SPDY1
chr2	73,144,811	73,145,197	386	12	0.00597692	EMX1_1
chr2	145,275,060	145,275,199	139	2	0.00815419	ZEB2_2
chr2	145,277,789	145,277,999	210	3	0.0152982	ZEB2_3
chr3	50,375,356	50,375,735	379	8	0.0376443	RASSF1_1
chr3	189,838,428	189,838,448	20	1	0.021121	LEPREL1
chr6	43,612,714	43,613,053	339	11	0.0128926	RSPH9
chr6	105,584,212	105,584,565	353	6	0.00688274	BVES
chr7	27,135,555	27,135,603	48	1	0.00489255	HOXA1_2
chr7	27,204,917	27,205,991	1074	42	0.0153748	HOXA9
chr8	41,166,680	41,167,113	433	14	0.0106293	SFRP1_2
chr9	21,974,883	21,975,014	131	1	0.00554111	CDKN2A_1
chr10	3,146,671	3,146,938	267	3	-0.0870769	PFKP_2
chr10	17,270,171	17,270,257	86	1	0.0128362	VIM_1
chr10	17,270,997	17,271,115	118	1	0.0060971	VIM_2
chr10	17,271,135	17,271,826	691	16	0.00889222	VIM_2
chr10	17,495,567	17,496,648	1081	17	0.00803994	ST8SIA6
chr12	4,383,371	4,383,543	172	2	0.00583131	CCND2
chr12	52,400,812	52,400,998	186	4	0.0178743	GRASP_1
chr14	21,493,902	21,494,093	191	2	0.0113818	NDRG2_2
chr14	54,421,257	54,421,685	428	8	0.00733723	BMP4_2
chr14	54,423,421	54,423,631	210	2	0.0134678	BMP4_3
chr16	56,701,912	56,702,180	268	10	0.0104061	MT1G
chr17	1,957,353	1,957,541	188	6	0.00675796	HIC1_1
chr17	6,616,866	6,616,867	1	1	0.0112864	SLC13A5
chr17	6,616,990	6,617,023	33	1	0.0105169	SLC13A5
chr17	41,363,727	41,364,199	472	7	0.0126908	TMEM106A
chr17	76,355,267	76,355,603	336	4	0.0366104	SOCS3
chr19	43,967,371	43,967,489	118	2	0.0137916	LYPD3_1
chr19	43,967,527	43,967,528	1	1	0.00287099	LYPD3_1
chr19	43,967,561	43,967,585	24	1	0.00641582	LYPD3_1
chr19	43,968,549	43,968,618	69	2	0.0289844	LYPD3_2
chr22	46,658,774	46,659,161	387	7	0.0169458	PKDREJ
chrX	30,326,466	30,326,947	481	4	− 0.0631399	NR0B1_1

MethPipe 4.1.1 program using default parameters of bin size 1:200:1 and CpG *p*-value of 0.01 was used to identify the differentially methylated regions.

### Validation of NGS-identified HCC-specific DMRs by MSqPCR assays

To validate these eight selected DMRs, an archived cohort of 144 patients (81 HCC and 63 non-HCC)^20^ (Table 3) was used. Due to availability of archived DNA, not all eight genes were assessed for all patients. Of the eight genes tested, the methylation of five genes, *GRASP (*m*GRASP)*, *CCND2 (mCCND2)*, *HOXA9 (mHOXA9)*, *BMP4 (mBMP4)* and *ECE1 (mECE1)*, were found to be significantly different (*p* < 0.05) between HCC and non-HCC patients. Methylation levels for non-HCC and HCC patients are listed in Supplementary Table 2 and 3, respectively. However, *mCCND2* was excluded from further assessment due to low incidence in HCC (7%, n = 29), while the other four (*mGRASP*, *mHOXA9*, *mBMP4*, and *mECE1)* genes had an HCC incidence ranging from 19 to 72%. These four potential markers were further tested in the control group (n = 11) of normal donors for specificity and were found to be undetectable (Table 3).

**Table 3:** Preliminary screening of eight MSqPCR targets in an archived urine cohort.

MSP Target (# HCC/Non-HCC)	Biomarker (+ / − )	HCC	Non-HCC	*p*-value (Fisher’s Exact)	Normal
		# (%)	# (%)		# (%)
SFRP1	+	3 (17%)	1 (6%)	0.602	–
(18/18)	−	15 (83%)	17 (94%)		
GRASP	+	12/21 (57%)	3 (16%)	0.010	0/11 (0%)
(21/19)	−	9/21 (43%)	16 (84%)		
CCND2	+	2 (7%)	6 (32%)	0.045	–
(29/19)	−	27 (93%)	13 (68%)		
HOXA9	+	7 (30%)	0 (0%)	0.030	0/11 (0%)
(23/14)	−	16 (70%)	14 (100%)		
BMP4	+	7 (19%)	0 (0%)	0.035	0/11 (0%)
(36/24)	−	29 (81%)	24 (100%)		
ECE1	+	18 (72%)	11 (42%)	0.048	0/11 (0%)
(25/26)	−	7 (28%)	15 (58%)		
VIM	+	4 (15%)	3 (25%)	0.654	–
(27/12)	−	23 (85%)	9 (75%)		
EMX1	+	1 (6%)	0 (0%)	1.000	–
(16/6)	−	15 (94%)	6 (100%)		

The cut-off for methylation levels is defined as any detectable level of methylation.

### Development of novel urinary methylated DNA markers for HCC screening

**Figure 2:**
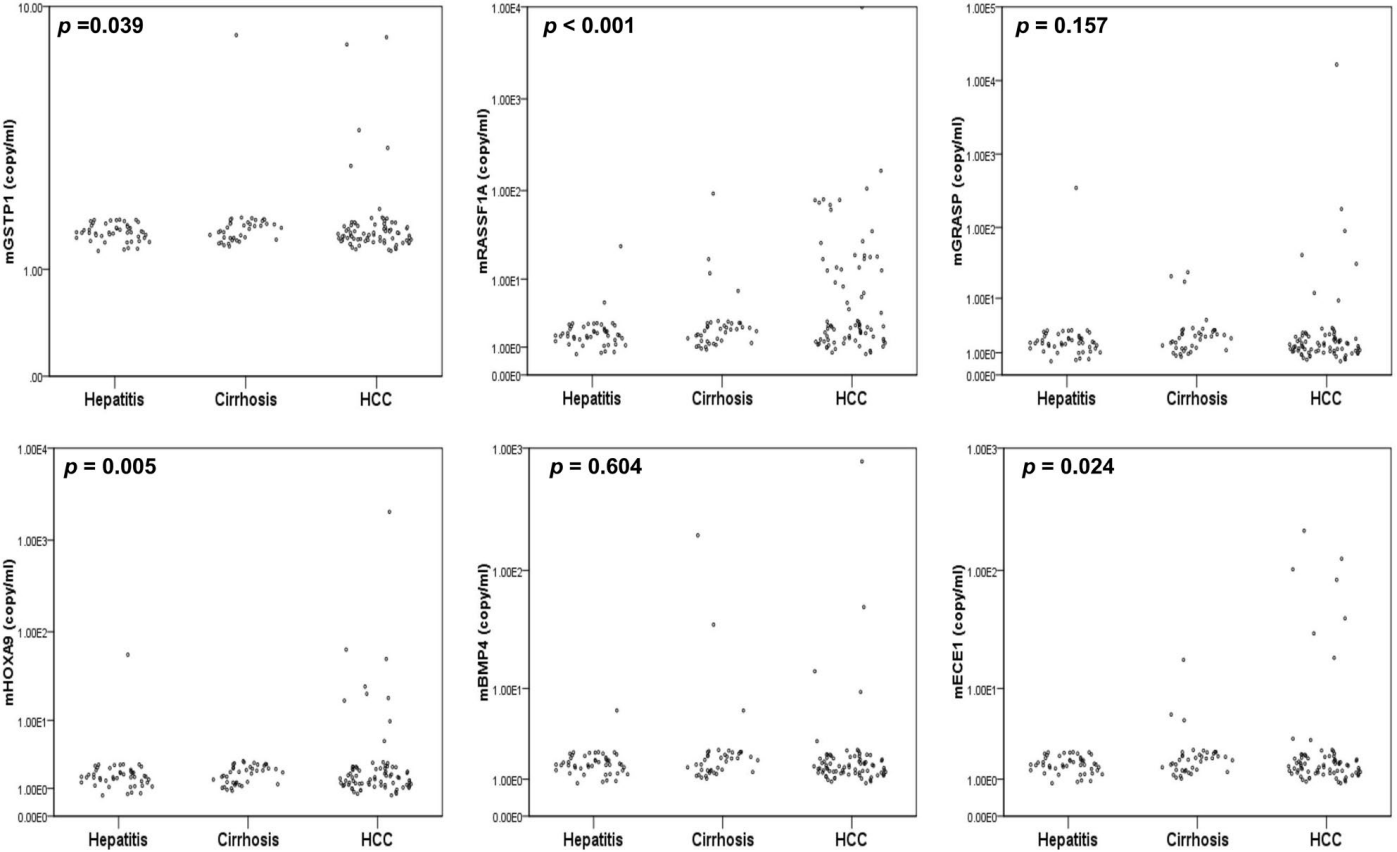
Methylation levels of DNA markers in urine from patients with HCC and non-HCC controls (hepatitis and cirrhosis). The methylation levels of each biomarker are shown in scatter plots by disease group and evaluated using the non-parametric independent samples Mann–Whitney U test comparing 78 HCC versus 87 non-HCC (hepatitis and cirrhosis).* p*-values are noted in each comparison.

Next, the performance of four urinary methylated genes *mGRASP*, m*HOXA9*, *mBMP4*, and *mECE1* genes together with the previously reported urine *mGSTP1* and *mRASSF1a* markers for distinguishing HCC from non-HCC by MSqPCR in urine of an independent cohort of 87 non-HCC (47 hepatitis and 40 cirrhosis), and 78 HCC patients (Table 4) was evaluated. The methylation levels of each candidate marker in each disease category are plotted in Fig. 2. Patients with HCC had significantly higher levels of *mRASSF1A* (*p* < 0.001), *mHOXA9* (*p* = 0.005), *mECE1* (*p* = 0.024), and *mGSTP1* (*p* = 0.039) in urine than those of non-HCC. No significant differences were seen in the levels of *mGRASP* (*p* = 0.157) and *mBMP4* (*p* = 0.604) in urine between HCC and non-HCC groups. Despite the low individual performances of *mGRASP* and *mBMP4*, it is possible, they may contribute to the performance in a marker panel. These two markers were included for marker panel development by using the backward selection method. ROC curves (Supplementary Fig. 3) were constructed for each individual marker and compared to serum AFP alone (Table 5) and in combination as a panel. As expected, of six markers evaluated, *mBMP4* exerted the lowest AUROC of 0.509 followed by *mGRASP* with AUROC of 0.522. For marker panel development, all six methylated genes and AFP were included in the logistic modeling, followed by exclusion of the least significant gene using the backward selection method. This was repeated until all included methylated genes were significant with respect to a cut-off of 0.3, chosen to obtain a target number of 3–5 biomarkers. As a result of the model selection, four markers, *mRASSF1a*, *mGRASP*, *mHOXA9*, and *mECE1* together as a 4-marker panel performed similar to a 6-marker panel as determined by the AUROC (Table 6 and Supplementary Fig. 4). Therefore, both the 6- and 4-marker panel in combination with serum AFP were assessed using a previous established Two-Stage model^28^. The AUROC of the 6- and 4-marker panel with AFP was 0.908 (95% CI, 0.8656–0.9252) and 0.907 (0.8627–0.9508), respectively (Table 6). This was significantly higher than AFP alone which had an AUROC 0.841 (6-marker, *p* = 0.0026; 4-marker, *p* = 0.0031).

**Table 4:** Patient clinical characteristics of the independent validation cohort in this study.

Diagnosis	Patient cohort (n = 165)		
	Hepatitis B (n = 47)	Cirrhosis (n = 40)	HCC (n = 78)
Median age (IQR range), years	55.6 (40–76)	59.2 (45–83)	65.2 (22–89)
Gender (M:F)	23:24	26:14	65:13
Etiology			
HBV	43	17	6
HCV	0	5	18
HBV/HCV	4	1	1
Non-viral	0	16	51
Unknown	0	1	2
BCLC Stage (n)			
0			4
A			23
B			35
C			14
D			2
AFP (ng/mL)			
< 20	47	36	55
≥ 20	0	4	23

BCLC: Barcelona Clinic Liver Cancer staging. AFP: alpha fetoprotein; HBV: Hepatitis B, HCV: Hepatitis C.

**Table 5:** AUROC of individual markers.

Predictor	AUROC (95% confidence interval)
*mGSTP1*	0.533 (0.501–0.564)
*mRASSF1A*	0.705 (0.642–0.769)
*mGRASP*	0.522 (0.486–0.581)
*mHOXA9*	0.559 (0.517–0.601)
*mBMP4*	0.509 (0.474–0.544)
*mECE1*	0.548 (0.506–0.590)
AFP (≥ 20 ng/mL)	0.841 (0.778–0.904)

**Table 6:** AUROC comparison of urine methylation gene panels.

Predictor	AUROC	Standard error	95% confidence interval		Sensitivity (%)	Specificity (%)
			Lower limit	Upper limit		
Serum AFP	0.841	0.032	0.7784	0.9036	29.5	95.4
6 mDNA	0.725	0.0357	0.6548	0.7946	47.4	89.7
4 mDNA	0.726	0.0345	0.6584	0.7935	47.4	92.0
AFP + 6 mDNA	0.908	0.0221	0.8656	0.9523	79.5	85.1
AFP + 4 mDNA	0.907	0.0225	0.8627	0.9508	79.5	86.2

4 mDNA, urine methylation (m) DNA biomarker panel which includes *mRASSF1A*, *mGRASP*, *mHOXA9*, and *mECE1.*

6 mDNA, mDNA biomarker panel which includes *mRASSF1A*, *mGSTP1*, *mGRASP*, *mHOXA9*, *mBMP4*, and *mECE1.*

**Figure 3:**
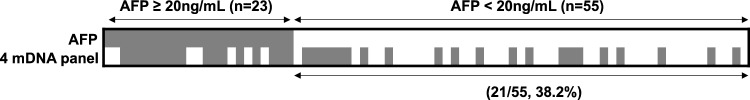
Distribution of HCC patients stratified by serum AFP cut-off of 20 ng/mL. The marker values are derived from the 4-marker urine methylation (4 mDNA) panel (*mRASSF1a, mGRASP*, *mHOXA9*, and *mECE1*). Each box represents a patient sample, where positive marker values detected are shaded in gray.

The sensitivities of the 6- and 4-gene panel combined with AFP were the same at 79.5%; however, the 4-gene panel with AFP displayed a slightly higher specificity at 86.2% compared to the 6-marker panel at 85.1%. Additionally, both the 6- and 4-marker panel alone detected 38% of AFP negative HCC that would otherwise be missed by AFP alone (Fig. 3). In general, the 6- and 4-marker panel in combination with AFP improved the detection sensitivity compared to AFP alone at a 20 ng/mL cutoff from 29.5% to 79.5% by Two-Stage model.

## Discussion

In this study, a HCC-targeted methylation NGS assay was developed to directly discover urinary methylated DNA genes for HCC screening in urine from a discovery cohort, with subsequent development of MsqPCR assays for 8 selected candidate genes for biomarker development in an independent cohort. To this end, aberrant methylation of four genes (m*GRASP, mHOXA9, mBMP4*, and m*ECE1*) were selected as potential urinary biomarkers for HCC screening. The addition of these newly identified urinary methylated DNA markers to our previously developed methylated DNA markers, *mRASSF1a* and *mGSTP1*, in combination with AFP showed an increased sensitivity from 30 to 80% for HCC screening as compared to AFP alone.

While others have applied MSqPCR to transrenal urine cfDNA based on known methylation markers^14,16,29,30^, this would be the first study to employ methyl-seq NGS for discovery of new methylation DNA markers directly from urine from HCC patients. Traditional liquid biopsy biomarker studies rely on tissue-informed studies to identify potential molecular targets where many markers may fail during validation screening. This can be partially attributed to biological differences between the tissue and liquid biopsy source (i.e. cell-free DNA)^26,31^, the survival of methylation markers in the circulation after apoptosis. Thus, in this study, we optimized a methyl-seq NGS assay for low DNA input and short, fragmented DNA (mostly less than 1-nucleosomal sized DNA), characteristic of transrenal DNA for biomarker discovery. Methyl-seq was performed directly in the body fluid of interest, that is intended to be used for a cancer screening test, in this case a urine test. Encouragingly, we demonstrated that promising markers were discovered by using methyl-seq, verified by MSqPCR, and validated in the validation set with statistical significance.

Interestingly, of the four verified methylated genes, three (*HOXA9, BMP4*, and *ECE1*) have reported associations with HCC^32–37^. *HOXA9* has a role in regulating gene expression and controlling functions related to morphogenesis and cell differentiation. *BMP4* belongs to the transforming growth factor-beta family and has been shown to impact cell growth, differentiation, migration, and invasion in cancer cells^34^. *ECE1* is a metalloprotease responsible for activating big endothelin-1 (ET-1), a potent vasoconstrictor and mitogen and plays a role in cancer-related properties such as uncontrolled proliferation and invasiveness through the activation of ET-1^38^. The role of the fourth gene *GRASP*, Grp-1 associated scaffold protein, has not been fully elucidated in HCC, but has been shown to play a role in cell migration^39^.

An independent patient cohort was used to assess the performance of the four novel methylation markers together with our two previously validated urinary HCC risk markers, *mGSTP1* and *mRASSF1a*. In this cohort, serum AFP alone has a sensitivity of 29.5% at a high specificity of 95% for detecting HCC at a cut-off of 20 ng/mL, as recommended by the AASLD. When a panel of 4 urine methylation markers plus serum AFP was evaluated, there was a significant improvement in HCC detection, detecting 50% more HCC, compared to using serum AFP alone. This highlights not only the potential application of new urinary HCC methylation markers for HCC screening, particularly for those low-AFP HCC patients, but also urine cfDNA as a viable source for epigenetic liquid biopsy.

It was encouraging to identify two known urinary HCC methylation markers, *mGSTP1* and m*RASSF1a* described in our previous reports^20^ by using our HCC-targeted methyl-seq method. *mRASSF1* was found to be significantly elevated in HCC by both comparisons: HCC *vs.* hepatitis (data not shown) and HCC *vs.* non-HCC (hepatitis + cirrhosis), as presented in Table 2. On the other hand, *mGSTP1* was found significantly elevated in HCC only when the comparison was performed between hepatitis and HCC (data not shown), likely due to the relative low incidence of HCC-associated *mGSTP1*.

Nonetheless, this validates the sensitivity of the methyl-seq approach to the discovery of methylation biomarkers in urine. Methylated DNA markers have also been studied in plasma cfDNA producing similar performance for early HCC detection (HelioLiver test^40^ and multitarget HCC blood test^41,42^). Interestingly, four urinary methylated biomarkers discovered in this study are different from the methylated DNA biomarkers included in plasma studies. In this study, a 4-methylation marker panel was identified and validated, while the HelioLiver test identified a 28-methylation marker panel targeting 77 CpG sites and the multitarget HCC blood^40^ includes two methylation markers (*HOXA1* and *TSPYL5*). The methyl-seq panel used in this urine biomarker study included *HOXA1* and *TSPYL5* genes, but they were not identified by our DMR analysis in our discovery cohort. As aberrant methylation of these two genes were shown to be promising blood biomarkers for HCC screening, of interest, MSqPCR assays for both *HOXA1* and *TSPYL5* genes were developed and tested in our archived urine DNA cohort. While methylation of both markers was found in our archived cohort by qPCR, they were found to have a low incidence (< 10%) in urine of HCC confirming the methyl-seq results (data not shown). It is possible that these two markers are not filtered or not preserved well in urine due to the increased presence of nucleases^43^.

To address the relatively small sample sizes in this biomarker discovery study, the performance of the discovered biomarkers was evaluated through two validation cohorts, an open-labeled cohort using archived specimens and an independent validation cohort. Encouragingly, the performance of the discovered biomarkers has been validated with statistical significance. An ongoing validation of this 4- and 6-methylation marker panel in a broader and larger independent patient population that takes into account different ethnicities, etiologies, and other clinicopathological variables is in progress.

There are other limitations to discovering methylated DNA biomarkers in urine using the methyl-seq NGS approach. First, the bisulfite conversion process is known to damage DNA, therefore the amount of DNA needed for both biomarker discovery by methyl-seq NGS and for NGS data confirmation by MSqPCR assay are at least 5–10 times more than what is needed for DNA mutation analysis. Second, the cost to perform methyl-seq NGS of 30 HCC and 31 non-HCC samples is significant. MSqPCR assays have been shown to provide comparable alternatives for the limited amount of DNA and are cost-effective for subsequent large biomarker validation and training studies. The results derived from this innovative approach for biomarker discovery are encouraging and have the potential to be applied to other cancers. It is known that methylated biomarkers are often not cancer specific^44^. Other cancers were not included in this HCC biomarker discovery study because the discovered markers will be used in a well-defined HCC at-risk population, patients with cirrhosis or chronic hepatitis B virus infection, to identify patients to undergo diagnosis by sophisticated MRI/CT imaging. The possibility of these methylated markers being derived from other cancers in this HCC at-risk population will be small and can be ruled out by MRI/CT imaging study. Other cancers will be included in a larger validation study to determine the specificity.

Urine presents advantages over blood-based liquid biopsies, as urine can be routinely collected in remote areas with large volumes and multiple follow-ups, requiring little technical expertise. The method of urine cfDNA isolation plays a critical role in obtaining high yields of ctDNA^45^. As we have previously demonstrated centrifugation for removal of cell debris can also deplete HCC ctDNA^46^. In this study, genetic *TP53* 249 T mutation was not included as it is found to be associated with HBV-HCC given the demographics of this HCC patient cohort which is mostly not HBV-related^47,48^. Overall, these results suggest that methylated transrenal ucfDNA markers have the potential to serve as a noninvasive and sensitive approach to increase HCC screening performance.

## Materials and methods

### Study subjects and samples

All patient urine samples used in this study were obtained with written informed consent. Heartland institutional review board (IRB) approved the study (project #171,201–173). The study was performed in accordance with Heartland IRB’s guidelines and regulations. Urine samples were collected from Thomas Jefferson University Hospital (Philadelphia, PA), The John Hopkins Hospital (Baltimore, MD), University of Pennsylvania Hospital (Philadelphia, PA), Buddhist Tzu Chi Medical Center (Hualien, Taiwan, ROC), and the National Cheng-Kung University Medical Center (Tainan, Taiwan, ROC) between April 2013 and July 2021.

Three patient cohorts were used in this study as outlined in the flowchart (Fig. 1). First, urine DNA isolated from 31 non-HCC (hepatitis/cirrhosis) and 30 HCC patients was used as a biomarker discovery cohort, as shown in Table 1. Next, previously isolated archived DNA^20^ was used for candidate methylation marker selection by MSqPCR with inclusion of 3 HCC patients belonging to the discovery cohort due to availability of DNA. Lastly, an independent training cohort (n = 165) was used, summarized in Table 2, independent of the discovery cohort. HCC is characterized by the AJCC (TNM) staging.

Normal donor urine collected from 8 females and 3 males aged 19–59 years old was used as a control cohort to establish a methylation baseline for the newly identify methylated targets.

### Urine DNA isolation

Urine collection was performed as described previously^45,46^. Briefly, urine (50 mL) was collected from subjects with no liquid uptake for at least 2 h and mixed with EDTA to a final EDTA concentration of 30–50 nM. A minimum of 30 ml urine was required for urine DNA isolation. Urine DNA isolation was performed using the JBS urine cfDNA isolation kit (JBS Science Inc., Doylestown, PA, catalog number 08872) without removal of cell-debris by centrifugation^46^ on the JPurX-S200 instrument (JBS) per manufacturer’s specification. Only urine samples that yielded a concentration of ≥ 1 ng/ml were included in the study.

### Methyl-seq library prep

The preparation of the methyl-seq library for urine cell-free DNA (ucfDNA) was performed with NEBNext enzymatic methyl-seq kits (New England Biolabs, Ipswich, MA, catalog number E7120S). Approximately 40 ng ucfDNA was used for each library preparation following manufacturer’s instructions. After end-repair ucfDNA was ligated to NEBNext EM-seq Adaptors. The ligation product was purified with magnetic beads followed by enzymatic oxidation. After another round of clean-up with magnetic beads, oxidized DNA fragments were denatured with formamide at 85 °C and subsequently underwent an enzymatic deamination reaction to convert unmethylated cytosines to uracils. Converted ucfDNA was cleaned up and amplified by PCR to add dual indexes as the final product of the bisulfite converted NGS library.

### Hybridization capture with a custom HCC methylation panel and NGS sequencing

A custom panel of DNA methylation capture probes of 76 genes (Supplementary Table 4) selected based on the literature review for the positive strand of human genomic DNA and hybridization kits (Integrated DNA Technologies (IDT), Coralville, IA, catalog number 1080584) was ordered from IDT. Hybridization capture was performed following the IDT protocol at 63.2 °C for overnight binding. A total of 500 ng of each library was used for up to 6 libraries per capture. After overnight hybridization of capture probes to library DNA, the capture reactions were incubated with streptavidin beads at 63.2 °C for 45 min. The beads were washed with IDT buffers following the protocol from IDT and then used as PCR template to amplify captured library DNA fragments on the beads with 2 × KAPA HiFi HotStart ReadyMix (Roche Diagnostics, Indianapolis, IN, catalog number KK2602). Library PCR products were assessed on TapeStation 4200 (Agilent Technologies, Santa Clara, CA) for size distribution and quantification. The library DNA product from methylation capture was subjected to duplex sequencing on a MiniSeq with 300-cycle sequencing kits (Illumina, San Diego, CA, catalog number FC-420-1003) following instructions from Illumina. The loading concentration was 1.1 pM. At least 20% spike-in of PhiX or any balanced DNA library with different indexes was spiked-in for each sequencing run.

### Methylation-specific quantitative PCR (MSqPCR)

Bisulfite (BS) treatment of patient ucfDNA was performed using the EZ DNA Methylation-Lightning™ Kit (Zymo Research, Irvine, CA, catalog number D5030) following manufacturer’s guidelines except for post-BS clean-up which was performed on the JPurX-S200 instrument using manufacturer’s specification for Bisulfite clean-up kit (JBS, catalog number 08878). Bisulfite converted DNA was quantified by a MSqPCR assay that was developed to target the methylated C’s that were not affected by bisulfite conversion. Eight identified gene regions underwent short amplicon (< 70 bp) assay design for fragmented ucfDNA. Within the identified region, forward and reverse primers (Tm < 60 °C) were designed. The total number of targeted CpG sites per assay and assay condition are summarized in Supplementary Table 1. The MSqPCR was performed using the LightCycler 480 real-time PCR system (Roche) and LightCycler 480 SYBR Green master kit (Roche, catalog number 04707516001). The reaction contained 1 × SYBR Green master mix, 1.0 µmol/L primers. Each assay was developed using human methylated bisulfite-converted DNA template (HMBS) (ZYMO, catalog number D5015) as standard positive DNA control and bisulfite converted normal human DNA (BS-HuDNA) as negative control. The PCR was performed under the following conditions detailed in Supplementary Table 1. Each assay was developed with a sensitivity for at least 3 methylated DNA copies. BS-HuDNA (negative control) was used for specificity control as shown in Supplementary Fig. 2.

### Data analysis

NGS data generated on the MiniSeq was demultiplexed with Bcl2fastq (Illumina) to generate fastq files. Using Bismark v0.21.0 (Babraham bioinformatics), fastq files were aligned to bisulfite converted human genomic sequence to generate BAM files. The BAM files were used for methylome construction and analysis to identify DMRs and CpGs using MethPipe 4.1.1 default conditions (*i.e.* bin size 1:200:1 and CpG *p*-value of 0.01) following instructions in the manual of this pipeline (Andrew Smith’s lab, University of South California). For the MSqPCR assay design, the methylated CpG sites were assessed using the MethPipe proportion table output which contains the individual CpG read counts of methylated and unmethylated reads.

Individual methylation marker values obtained in the independent training cohort were depicted in a scatter plot and the non-parametric independent samples Mann–Whitney *U-*test was used to calculate the p-value for comparison between the HCC and non-HCC group due to the skewed distribution of the data. To evaluate the performance of the methylation panel to distinguish HCC from non-HCC, area under the receiver operating characteristic (AUROC) curves were constructed for each individual urine marker and AFP. A two-stage logistic regression model as previously described^20,28^ was used to assess the performance of 6- urine methylation marker panel alone and in combination with AFP. A 4-marker panel was obtained using the backward selection method to determine the least number of biomarkers for a similar performance to that of the 6-marker panel.

### Supplementary Information

Supplementary Information.

## Data Availability

The data generated or analyzed during this study are available from the corresponding author upon reasonable request.
