# Supplementary material for: Novel urine cell-free DNA methylation markers for hepatocellular carcinoma
Source: Sci Rep. 2023 Dec 7;13:21585. doi: 10.1038/s41598-023-48500-y (PMC10703769; doi:10.1038/s41598-023-48500-y)
Supplement: Supplementary file 1 — Supplementary Information. [file 41598_2023_48500_MOESM1_ESM.docx]

**Novel urine cell-free DNA methylation markers for hepatocellular carcinoma**

Selena Y. Lin^1^, Wei Xia^1^, Amy K. Kim^2^, Dion Chen^1, 3^, Shelby Schleyer^1^, Lin Choi^1^, Zhili Wang^1^, James P. Hamilton^2^, Harry Luu^2^, Hie-Won Hann^4^, Ting-Tsung Chang^5^, Chi-Tan Hu^6^, Abashai Woodard^7^, Terence P. Gade^7^, Ying-Hsiu Su^8^*

^1^JBS Science, Inc., Doylestown, PA, USA

^2^Division of Gastroenterology and Hepatology, Department of Medicine, The Johns Hopkins University School of Medicine, Baltimore, MD, USA

^3^ClinPharma Consulting, Inc., Phoenixville, PA, USA

^4^Department of Medicine, Division of Gastroenterology and Hepatology, Thomas Jefferson University Hospital, Philadelphia, PA, USA

^5^Department of Internal Medicine, National Cheng Kung University Hospital, College of Medicine, National Cheng Kung University, Tainan, Taiwan

^6^Division of Gastroenterology, Department of Internal Medicine, Hualien Tzu-Chi Hospital, Buddhist Tzu-Chi Medical Foundation, Hualien, Taiwan

^7^Department of Radiology, University of Pennsylvania College of Medicine, Philadelphia, PA, USA

^8^The Baruch S. Blumberg Institute, Doylestown, PA, USA

**Correspondence**: Ying-Hsiu Su, The Baruch S. Blumberg Research Institute, 3805 Old Easton Rd, Doylestown, PA, USA 18902; Tel: 215-489-4949; Fax: 215-489-4920; E-mail: [Ying-hsiu.su@bblumberg.org](mailto:Ying-hsiu.su@bblumberg.org).

# SUPPLEMENTARY FIGURES

**
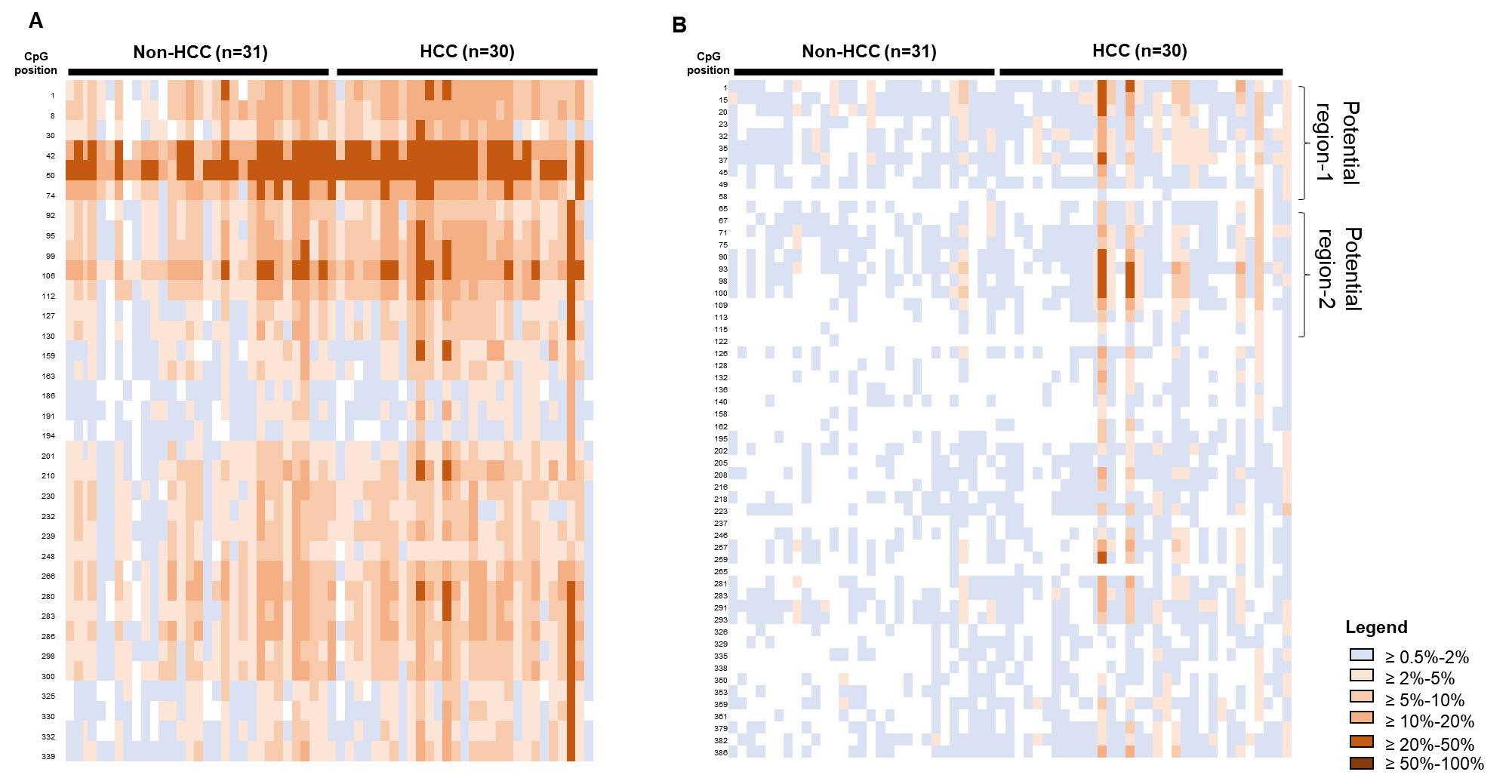
**

**Supplementary Figure 1. A) RSPH9 DMR (339bp) and B) EMX1 DMR region (386bp) identified in pilot cohort.** Differentially methylated region (DMR) of 339bp identified in RSPH9 (A) and EMX1 (B) are shown for all patients where CpG positions are numbered starting from position 1 as defined by MethPipe. The methylation % is calculated by the total read counts of methylation vs unmethylated reads and is indicated for each CpG site per patient by the color scheme in the figure legend.

**Supplementary Figure 2. Development of eight methylation-specific quantitative PCR (MS-qPCR).** Each quantitative assay was developed using ZYMO’s DNA control human methylated bisulfite DNA (HMBS) as a standard control and bisulfite-converted normal human DNA (BS-HuDNA). Amplification, standard, and melting curves are shown for each biomarker. Efficiency denotes qPCR run efficiency where highest quality of 2 would indicate number of target molecules doubling with every PCR cycle. Slope denotes PCR amplification kinetics of the standard curve, where a slope of -3.3 indicates perfect amplification.

**Supplementary Figure 3. Receiver-operating curves (ROC) of individual markers.** The area under the ROC (AUROC) are displayed for serum AFP and individual urine methylation DNA markers.

**Supplementary Figure 4. Receiver-operating curves (ROC) of urine methylation gene panels.** The area under the ROC (AUROC) are displayed for urine methylation biomarker panels with and without serum AFP (≥20ng/mL). 6 mDNA panel includes *mRASSF1A*, *mGSTP1*, *mGRASP*, *mHOXA9*, *mBMP4*, and *mECE1*. 4 mDNA panel includes *mRASSF1A*, *mGRASP*, *mHOXA9*, and *mECE1*.

# SUPPLEMENTARY TABLES

**Supplementary Table 1. MS-qPCR assay conditions.**

| **Assays** | **Target amplicon size (# of CpGs)** | **PCR conditions** |
| --- | --- | --- |
| *EMX1* | 57 bp (7) | 95˚C - 5mins (95˚C 10sec, 63˚C 10sec, 72˚C* 10 sec) x 45 cycles, melt |
|  |  |  |
| *VIM* | 50 bp (7) | 95˚C - 5mins (95˚C 10sec, 62˚C 10sec, 72˚C* 10 sec) x 45 cycles, melt |
|  |  |  |
| *ECE1* | 52 bp (7) | 95˚C - 5mins (95˚C 10sec, 61˚C 10sec, 72˚C* 10 sec) x 45 cycles, melt |
|  |  |  |
| *HOXA9* | 44 bp (6) | 95˚C - 5mins (95˚C 10sec, 58˚C 10sec, 72˚C* 10 sec) x 45 cycles, melt |
|  |  |  |
| *CCND2* | 50 bp (6) |  |
|  |  |  |
| *SFRP* | 42 bp (7) | 95˚C - 5mins (95˚C 10sec, 56˚C 10sec, 72˚C* 10 sec) x 45 cycles, melt |
|  |  |  |
| *GRASP* | 48 bp (6) |  |
|  |  |  |
| *BMP4* | 64 bp (7) | 95˚C - 5mins (95C 10sec, 54˚C 10sec, 72˚C* 10 sec) x 45 cycles, melt |
|  |  |  |

**Supplementary Table 2. Methylation levels in eight MSqPCR targets in a preliminary screening of a non-HCC cohort using archived DNA samples.**

| **# of Non-HCC^** | ***mSRPF1* (copy/ml)** | ***mGRASP* (copy/ml)** | ***mCCND2* (copy/ml)** | ***mHOXA9* (copy/ml)** | ***mBMP4* (copy/ml)** | ***mECE1* (copy/ml)** | ***mVIM* (copy/ml)** | ***mEMX1* (copy/ml)** |
| --- | --- | --- | --- | --- | --- | --- | --- | --- |
| 1 | 1.9 | 3.8 | 4.8 | BLOD | BLOD | 3.2 | 4.2 | BLOD |
| 2 | BLOD | 9.7 | 4.8 | BLOD | BLOD | 4.9 | 5.3 | BLOD |
| 3 | BLOD | 12.3 | 7.1 | BLOD | BLOD | 5.5 | 25.9 | BLOD |
| 4 | BLOD | BLOD | 18.4 | BLOD | BLOD | 5.8 | BLOD | BLOD |
| 5 | BLOD | BLOD | 21.5 | BLOD | BLOD | 9.1 | BLOD | BLOD |
| 6 | BLOD | BLOD | 26.2 | BLOD | BLOD | 14.5 | BLOD | BLOD |
| 7 | BLOD | BLOD | BLOD | BLOD | BLOD | 38.8 | BLOD |  |
| 8 | BLOD | BLOD | BLOD | BLOD | BLOD | 44.3 | BLOD |  |
| 9 | BLOD | BLOD | BLOD | BLOD | BLOD | 45.5 | BLOD |  |
| 10 | BLOD | BLOD | BLOD | BLOD | BLOD | 97.9 | BLOD |  |
| 11 | BLOD | BLOD | BLOD | BLOD | BLOD | 135.0 | BLOD |  |
| 12 | BLOD | BLOD | BLOD | BLOD | BLOD | BLOD | BLOD |  |
| 13 | BLOD | BLOD | BLOD | BLOD | BLOD | BLOD |  |  |
| 14 | BLOD | BLOD | BLOD | BLOD | BLOD | BLOD |  |  |
| 15 | BLOD | BLOD | BLOD |  | BLOD | BLOD |  |  |
| 16 | BLOD | BLOD | BLOD |  | BLOD | BLOD |  |  |
| 17 | BLOD | BLOD | BLOD |  | BLOD | BLOD |  |  |
| 18 | BLOD | BLOD | BLOD |  | BLOD | BLOD |  |  |
| 19 |  | BLOD | BLOD |  | BLOD | BLOD |  |  |
| 20 |  |  |  |  | BLOD | BLOD |  |  |
| 21 |  |  |  |  | BLOD | BLOD |  |  |
| 22 |  |  |  |  | BLOD | BLOD |  |  |
| 23 |  |  |  |  | BLOD | BLOD |  |  |
| 24 |  |  |  |  | BLOD | BLOD |  |  |
| 25 |  |  |  |  |  | BLOD |  |  |
| 26 |  |  |  |  |  | BLOD |  |  |

^, Number of samples tested for each marker were not overlapping. For instance, the first sample for each marker could be from different patients.

**Supplementary Table 3. Methylation levels in eight MSqPCR targets in a preliminary screening of a HCC cohort using archived DNA samples.**

| **# of HCC^** | **mSRPF1 (copy/ml)** | ***mGRASP* (copy/ml)** | ***mCCND2* (copy/ml)** | ***mHOXA9* (copy/ml)** | ***mBMP4* (copy/ml)** | ***mECE1***  **(copy/ml)** | ***mVIM* (copy/ml)** | ***mEMX1* (copy/ml)** |
| --- | --- | --- | --- | --- | --- | --- | --- | --- |
| 1 | 2.1 | 2.1 | 18.8 | 1.0 | 4.9 | 4.1 | 1.4 | 273.9 |
| 2 | 26.3 | 2.5 | 43.6 | 1.5 | 5.9 | 4.4 | 3.1 | BLOD |
| 3 | 8976.0 | 3.1 | BLOD | 3.3 | 6.6 | 7.5 | 1427.1 | BLOD |
| 4 | BLOD | 3.1 | BLOD | 3.8 | 11.9 | 8.5 | 16599.0 | BLOD |
| 5 | BLOD | 3.6 | BLOD | 7.5 | 37.2 | 9.9 | BLOD | BLOD |
| 6 | BLOD | 4.8 | BLOD | 23.9 | 66.0 | 13.5 | BLOD | BLOD |
| 7 | BLOD | 5.3 | BLOD | 2948.0 | 574.2 | 13.9 | BLOD | BLOD |
| 8 | BLOD | 5.6 | BLOD | BLOD | BLOD | 23.7 | BLOD | BLOD |
| 9 | BLOD | 7.3 | BLOD | BLOD | BLOD | 53.7 | BLOD | BLOD |
| 10 | BLOD | 15.3 | BLOD | BLOD | BLOD | 80.4 | BLOD | BLOD |
| 11 | BLOD | 19.1 | BLOD | BLOD | BLOD | 81.0 | BLOD | BLOD |
| 12 | BLOD | 26.3 | BLOD | BLOD | BLOD | 82.3 | BLOD | BLOD |
| 13 | BLOD | BLOD | BLOD | BLOD | BLOD | 165.6 | BLOD | BLOD |
| 14 | BLOD | BLOD | BLOD | BLOD | BLOD | 179.3 | BLOD | BLOD |
| 15 | BLOD | BLOD | BLOD | BLOD | BLOD | 224.8 | BLOD | BLOD |
| 16 | BLOD | BLOD | BLOD | BLOD | BLOD | 253.2 | BLOD | BLOD |
| 17 | BLOD | BLOD | BLOD | BLOD | BLOD | 476.7 | BLOD |  |
| 18 | BLOD | BLOD | BLOD | BLOD | BLOD | 1000.0 | BLOD |  |
| 19 |  | BLOD | BLOD | BLOD | BLOD | BLOD | BLOD |  |
| 20 |  | BLOD | BLOD | BLOD | BLOD | BLOD | BLOD |  |
| 21 |  | BLOD | BLOD | BLOD | BLOD | BLOD | BLOD |  |
| 22 |  |  | BLOD | BLOD | BLOD | BLOD | BLOD |  |
| 23 |  |  | BLOD | BLOD | BLOD | BLOD | BLOD |  |
| 24 |  |  | BLOD |  | BLOD | BLOD | BLOD |  |
| 25 |  |  | BLOD |  | BLOD | BLOD | BLOD |  |
| 26 |  |  | BLOD |  | BLOD |  | BLOD |  |
| 27 |  |  | BLOD |  | BLOD |  | BLOD |  |
| 28 |  |  | BLOD |  | BLOD |  |  |  |
| 29 |  |  | BLOD |  | BLOD |  |  |  |
| 30 |  |  |  |  | BLOD |  |  |  |
| 31 |  |  |  |  | BLOD |  |  |  |
| 32 |  |  |  |  | BLOD |  |  |  |
| 33 |  |  |  |  | BLOD |  |  |  |
| 34 |  |  |  |  | BLOD |  |  |  |
| 35 |  |  |  |  | BLOD |  |  |  |
| 36 |  |  |  |  | BLOD |  |  |  |

^, Number of samples tested for each marker were not overlapping. For instance, the first sample for each marker could be from different patients.

**Supplementary Table 4. Methyl-seq panel list of 76 genes.**

| *AK055957* | *FBP1* | *NKX6-2* | *ST20* |
| --- | --- | --- | --- |
| *AKAP12* | *FHIT* | *NPY1R* | *ST8SIA6* |
| *APC* | *FOXD2* | *NR0B1* | *STEAP4* |
| *BMP4* | *GRASP* | *NXPE3* | *TBX2* |
| *BVES* | *GSTP1* | *OXGR1* | *TFPI2* |
| *CCDC37* | *HHIP* | *PFKP* | *TIMP3* |
| *CCND2* | *HIC1* | *PKDREJ* | *TMEM106A* |
| *CCNJ* | *HIST1H3E* | *PRDM2* | *TSLP* |
| *CDH1* | *HOXA1* | *PTGS2* | *TSPYL5* |
| *CDKL2* | *HOXA9* | *RASSF1* | *VIM* |
| *CDKN2A* | *KCNC1* | *RBAK* | *WIF1* |
| *CDKN2B* | *LEPREL1* | *RSPH9* | *ZEB2* |
| *CFTR* | *LOC255167* | *RUNX3* | *ZFP41* |
| *CLEC11A* | *LYPD3* | *SFRP1* | *ZMYND10* |
| *COL25A1* | *MAP9* | *SLC13A5* | *ZNF154* |
| *CPS1* | *MGMT* | *SOCS1* | *ZNF540* |
| *DAB2IP* | *MT1E* | *SOCS3* |  |
| *DNM3* | *MT1G* | *SPDY1* |  |
| *ECE1* | *MT1M* | *SPINT2* |  |
| *EMX1* | *NDRG2* | *SRD5A2* |  |
